# Ecological Network Analysis for Economic Systems: Growth and Development and Implications for Sustainable Development

**DOI:** 10.1371/journal.pone.0100923

**Published:** 2014-06-30

**Authors:** Jiali Huang, Robert E. Ulanowicz

**Affiliations:** 1 Centre of Mountain Development, Institute of Mountain Hazards and Environment, Chinese Academy of Sciences, Chengdu, Sichuan, China; 2 Chesapeake Biological Laboratory, University of Maryland, Solomons, Maryland, United States of America; 3 Arthur R. Marshall Laboratory, University of Florida, Gainesville, Florida, United States of America; Cinvestav-Merida, Mexico

## Abstract

The quantification of growth and development is an important issue in economics, because these phenomena are closely related to sustainability. We address growth and development from a network perspective in which economic systems are represented as flow networks and analyzed using ecological network analysis (ENA). The Beijing economic system is used as a case study and 11 input–output (I-O) tables for 1985–2010 are converted into currency networks. ENA is used to calculate system-level indices to quantify the growth and development of Beijing. The contributions of each direct flow toward growth and development in 2010 are calculated and their implications for sustainable development are discussed. The results show that during 1985–2010, growth was the main attribute of the Beijing economic system. Although the system grew exponentially, its development fluctuated within only a small range. The results suggest that system ascendency should be increased in order to favor more sustainable development. Ascendency can be augmented in two ways: (1) strengthen those pathways with positive contributions to increasing ascendency and (2) weaken those with negative effects.

## Introduction

Growth and development are fundamental processes in systems ranging from cells to economic systems and even the universe. The quantification of growth and development is a necessary prerequisite to any treatment of sustainable development. In the fields of economics, increases in GDP or per capita GDP commonly are used to represent economic growth [1]–[6]. Sometimes the very same indicators are used to assess economic development [7]–[10], even though economic development is distinct from economic growth [11]. Such conflation indicates that the differences between economic growth and development remain fuzzy, and the confusion often is repeated in quantitative treatments. In this study, we use information-based ecological network analysis (ENA) indicators to quantify economic growth and development. Our approach has two advantages: First, the separate meanings of economic growth and development can be clearly distinguished. Secondly, the combined action of growth and development can be quantified in terms of a single index.

ENA is a systems-oriented method used to identify holistic properties, especially those not evident through direct observation, by focusing upon *interactions* among the components of the system [12]–[15]. In one of the first ENA studies, Hannon (1973) imported input–output methods from economics into ecosystems. Since then, ENA has been used widely in the field of ecology [16]–[21]. Increasingly it is being adopted for other applications, such as city management [22], [23] and water systems [24]–[27]. ENA has been re-imported back into economics, mainly as a form of general qualitative analyses of problems such as the causes of and solutions to banking crises [28], [29] and how to quantify economic sustainability from a network perspective[30], [31]. ENA, however, is rarely used to investigate the specific structures of economic systems of interest [32].

In ecology, ENA addresses the relationships between the individual compartments and the overall properties of a system by analyzing material–energy–information networks. Similar concepts and network methods can be applied, however, to all matter–energy–information flow systems in general, because of widespread and significant parallels among behavioral patterns and development dynamics in various fields [30]. In particular, ENA can be utilized for both ecological and economic systems. The first step in ENA is to construct a flow network that represents the system of interest. Over 60 years ago, Leontief showed that economic structure can be effectively modeled as an input–output map of goods, services, money, or value circulating within and across a network of businesses [33]. Thus, we focus upon currency networks, which readily can be constructed from I-O tables, to represent our economic systems of interest.

From a flow network perspective, growth usually implies increase and/or expansion, which may involve either a greater spatial extent or the accretion of flow medium. Growth is commonly quantified as any increase in total system throughput (*TST*) [34]. The terminology introduced here, and the indexes *AMI, A*, *C* and *R* will be defined quantitatively below. Development, however, implies an increase in organization, which is independent of growth and can be represented by an increase in the average mutual information (*AMI*) inherent in the network [34]. The combined actions of growth and development, as they pertain to increases in network size and organization, will be represented as an increase in system ascendency (*A*) [34], [35]. By calculating indicators of *TST*, *AMI*, *A*, and the development capacity (*C*) [34], we can obtain a useful picture of economic growth and development as a prelude to studying the factors that account for the observed phenomenon. We should also be able to identify means by which to enhance sustainable development.

The objectives of this study are threefold: (1) to provide an example of the potential of ENA for diagnosing economic systems, using as a case study the economy of Beijing from 1985 to 2010; (2) to describe the growth and development of that system from a currency network perspective; and (3) to propose suggestions for approaching sustainable development by balancing ascendency (*A*) with resilience (*R*).

## Methodology

### 2.1. Study area

Beijing is the capital and economic center of China. It recorded the second highest GDP among all the cities in China from 1985 to 2010, following only Shanghai [36]–[38]. Its constant-price GDP (in terms of 1985 ¥) increased from ¥25.7 billion to ¥326.3 billion over the designated period [36]. This statistic only partly describes the growth of the Beijing economy, however. During 1985–2010, there were structural changes as well. The main changes were that the proportion of industry output to the total output decreased from 59.0% to 31.5% and other services increased from 13.8% to 37.9% according to I-O tables. Changes in GDP and industry structure, nevertheless, cannot adequately reflect the key factors boosting growth and development. Analysis of economic growth and development using ENA, by contrast, can provide further insight into this issue.

### 2.2. Calculation of growth

*TST* is the sum of all flows in a system and is a measure of the rate at which medium is being processed by the system. Thus, like GDP, it reflects the size and overall activity of the system. We define 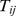 as the flow of medium from compartment 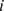 to compartment 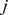. Using the notation whereby a subscript dot in the place of a subscript indicates summation over that index, 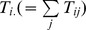 represents the rate at which medium is leaving compartment 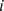 during a time interval, while 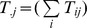 represents the rate of all inputs to compartment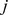 during the same interval. In particular, 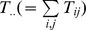 represents the total activity of a system. *TST* is thus expressed as
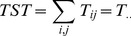
(1)


Growth, in economic terms, usually refers to the increase in total exchanges of flow medium [34]. Whence, growth can be characterized as any increase in *TST*.

### 2.3. Calculation of development

Development is defined as an increase in organization, which is independent of the size of a system [34]. For a highly organized system, if it is known that a flow has left compartment 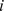 at time 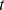, then a great deal of information about which compartment 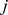 will receive the flow at time 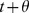 is inherent in its flow structure (network). The more organized a system is, the more information it can provide. An ecological information-based index called the average mutual information (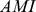) is introduced to estimate such organization, so that any increase in 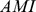 becomes a measure of development.

Information is defined as a decrease in indeterminacy. If the probability of the outcome of event 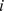 is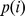, the indeterminacy of event 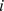 is gauged by 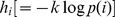 [39]. The usual convention is that 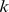 defines the unit of information related variables. The value of 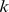 is determined by the base of the logarithm. For example, if the base of the logarithm is 2, 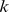 is 1 “bit”. If natural logarithms are used, 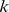 is 1 “nat”. The average indeterminacy for the whole system then becomes
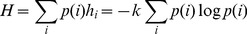
(2)


If one knows both the indeterminacy of event 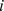, and the indeterminacy of event 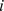 whenever event 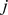 occurs, then the decrease in indeterminacy for event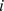 induced by event 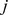 can be calculated by difference as 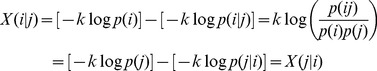
(3)


The symmetry in Eq. (3) implies that the information that event 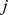 provides regarding event 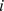 is equal to the information that event 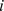 provides regarding event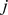. Hence, Eq. (3) captures the mutual information that events 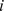 and 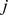 provide about each other. The 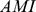 of the whole system can be obtained by multiplying each 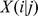 by its corresponding joint probability and summing over all combinations of events.
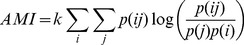
(4)


In flow networks, events 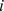 and 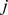 can be regarded as flows leaving compartment 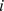 and flows entering compartment 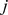, respectively, at time 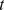. The joint event 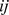 represents a flow leaving compartment 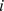 and entering compartment 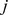. These identifications allow us to estimate the probabilities of these events in terms of the observed flows as,
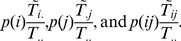
(5)


Substituting these estimators in Eqs. (2) and (4), yields
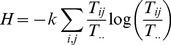
(6)and



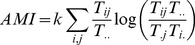
(7)The convexity of the logarithmic function guarantees that 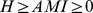 [21], [34]. Thus, 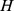 serves as an upper bound on *AMI*.

*AMI* quantifies the amount of flow diversity (*H*) that is encumbered by structural constraints. It quantifies regular, orderly, coherent and efficient behaviors in a system. Higher *AMI* values reflect tighter constraints on the movement of medium. Such structures are also said to be highly organized. If certain links are more efficient in transferring medium, they will become increasingly important, so that an ever greater amount of medium passes through them and they progressively predominate over less efficient links. This dynamic is the consequence of a positive feedback that selects a few efficient links and prunes away less efficient links, potentially yielding a structure composed of a few links. Due to positive feedback, systems develop in the direction of a more organized structure of exchanges. Thus, an increase in *AMI* corresponds to an increase in system organization, i.e., development.

### 2.4. Calculation of growth and development

The product of 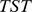 with 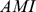 is defined as ascendency [34]. Ascendency, which combines system activity and organization, provides a single measure of the unitary process of growth and development.
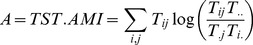
(8)


Scaling 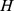 by 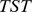 yields the development capacity of a system, which serves as an upper bound on system ascendency. 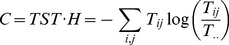
(9)


The difference between development capacity and ascendency can be regarded as resilience, an attribute that is complementary (opposite) to ascendency.
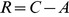
(10)


### 2.5. Complete decomposition model

Complete decomposition of any compound variable *V,* gives rise to a residual that can be evaluated according to the assumption of ‘jointly created and equally distributed’ [40]. So if 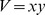, i.e. the variable 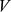 is determined by factors *x* and *y*, during the time interval 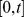, then the change in 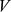 can be calculated in terms of its constituent factors as
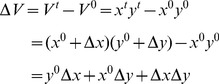
(11)where 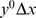 and 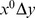 are the contributions of changes in 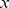 and 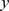, respectively, to the total change in 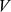. The term 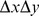 is the residual. Assuming that *x* and *y* contribute equally to 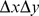, the contributions of factors *x* and *y* to 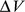 become:



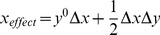
(12)




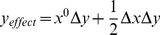
(13)


Below we will use this result to analyze the impact of 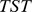 and 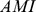 on ascendency.

### 2.6. Data sources

To analyze the growth and development of Beijing using ENA, it is necessary first to construct the appropriate currency networks. We pointed out in Section 1 that I-O tables can be readily converted into currency networks. I-O tables of Beijing in 1985, 1987, 1990, 1992, 1995, 1997, 2000, 2002, 2005, 2007 and 2010 were used in this study, and taken from the Beijing Municipal Bureau of Statistics, where these data were complied and published. The 1985 I-O table is made up of 68 sectors. The I-O tables for 1987, 1990, 1992 and 1995 are made up of 33 sectors. Those for 1997 and 2000 consist of 40 sectors, and those for 2002, 2005, 2007 and 2010, of 42 sectors. For purposes of analysis and comparison, the I-O data were aggregated into six common sectors according to the National Bureau of Statistics of China classification: agriculture; industry; construction; commercial and catering trade; transportation, post, and telecommunications; and other services.

### 2.7. Construction of currency networks

**Figure 1:**
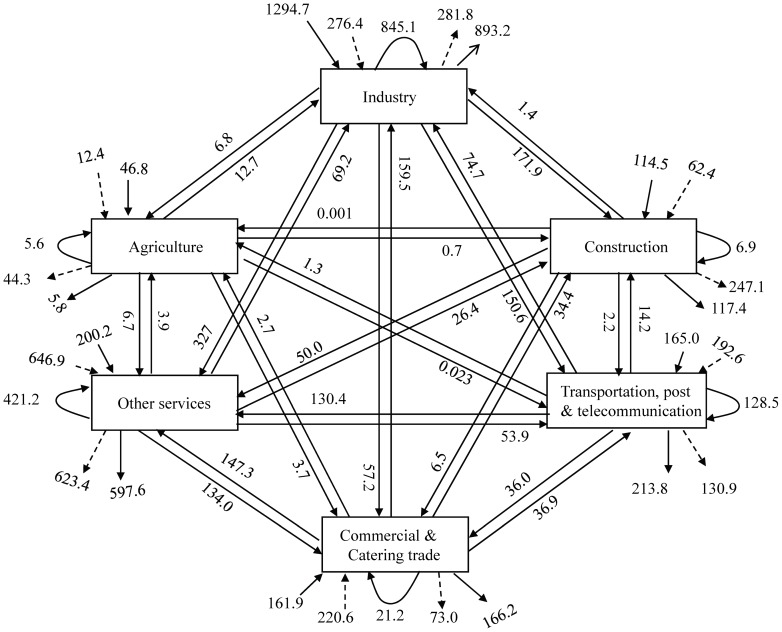
Currency flows among sectors of the Beijing economy (¥ billions y^−1^) in 2010. Solid arrows not originating from a box represent inputs from external systems and broken lines represent value-added capital. Solid arrows not terminating in a box represent outputs to other systems and broken lines represent end use. Arrows forming arcs represent inputs from the sector itself. Other arrows represent currency flows among sectors.

Currency networks consist of sectors (nodes) and currency exchanges (flows). Both categories must be well-defined. The sectors have already been identified. The practice in thermodynamics (and one maintained in ecology) is to classify flows into four categories: (1) exchanges among nodes within the system; (2) inputs of medium from outside the system; (3) exports of usable medium to other systems; and (4) exports of dissipated medium of no use to other systems [21], [34]. Whether or not this approach can be applied to currency networks, however, remains an open question. In economics, flows always exist in opposite directions: commodities flow one way and currencies for those commodities flow in the opposite direction. Currency networks, however, do not simply mirror physical flows. In currency networks, for example, value-added capital is a type of input for which there is no physical counterpart. Hence, the four-category scheme of thermodynamics may not be appropriate for classifying currency flows. As long as the crucial distinction between inputs and outputs is maintained, however, the definitions of key variables, such as *TST*, *AMI*, and *A*, are not qualitatively affected by the various sub-distinctions made among inputs and outputs [34]. Accordingly, we classified currency flows into five categories: (1) flows among sectors; (2) inputs from outside the system; (3) outputs to other systems; (4) value-added capital for each sector; and (5) the end use of each sector. The first three currency flows correspond to the first three ecological flows. Figure 1 depicts a currency network constructed from I-O tables for Beijing in 2010. Other I-O tables were used to estimate currency networks in the same way. In all, 11 currency networks were constructed.

## Results and Discussion

### 3.1. TST trend and analysis of growth

**Figure 2:**
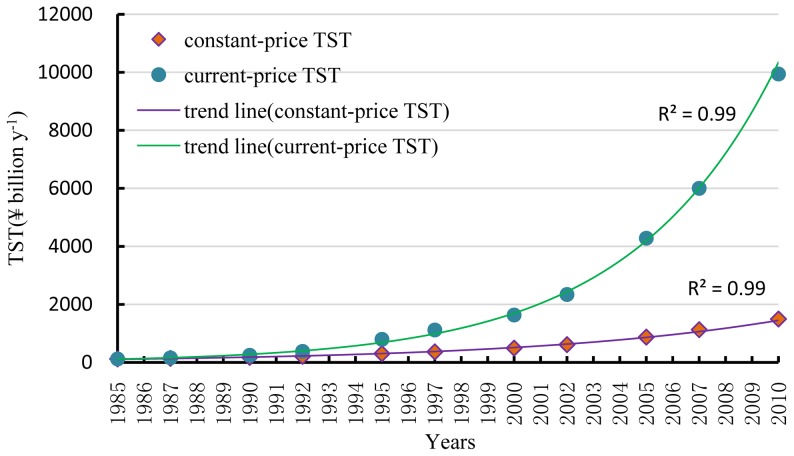
History of *TST* for Beijing, 1985–2010.

I-O tables between 1985 and 2010 were converted into currency networks and values of *TST* calculated according to Eq. (1) were obtained for each year. Each magnitude of *TST* was converted into its constant-price equivalent via the GDP index. The base year was taken as 1985, and the adjusted *TST*s are depicted in Figure 2.

Figure 2 reveals that current-price *TST* increased exponentially from ¥118 billion y^−1^ in 1985 to ¥9940 billion y^−1^ in 2010, with exponential growth rate 19.41%. The constant-price *TST* increased exponentially from ¥118 billion y^−1^ to ¥1496 billion y^−1^, with a lower exponential growth rate 10.70%. The slower rate may reflect that inflation has become progressively worse during those years. In either case, the economic system of Beijing grew exponentially during the study period. One of the main reasons for this fast growth is likely the market reform policy introduced in 1978.

### 3.2. The trend in AMI and analysis of development

**Figure 3:**
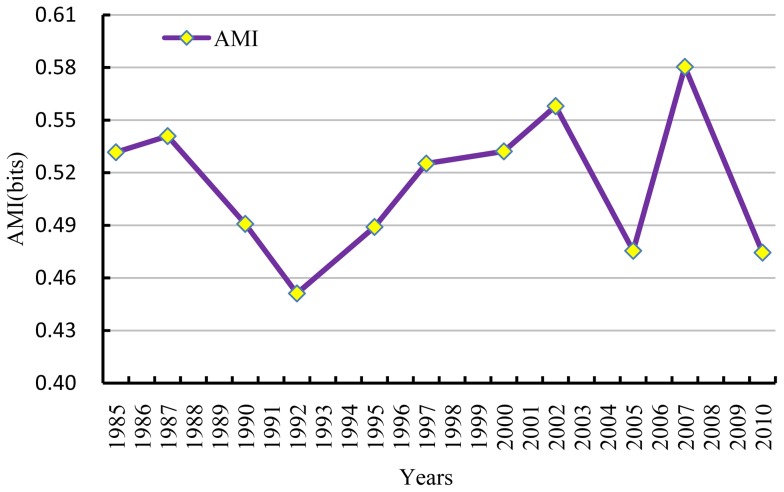
*AMI* of currency flows in Beijing from 1985–2010.

We substituted flow data for each currency network into Eq. (7) to derive an AMI for each year. The results are depicted in Figure 3.

Figure 3 shows that *AMI* fluctuated between 0.45 and 0.58 bits over the study interval. The fluctuations in *AMI* can be interpreted in various ways, but they mainly reflect the influence of global economic turbulence upon the Beijing economy. In particular, the decrease in *AMI* from 1987 to 1992 may be due to the recession of the 1990s (e.g., Black Monday, 1987). Similarly, the decrease in *AMI* from 2007 to 2010 may reflect the influence of the subprime lending crisis in the USA during 2008. In these years, the sudden decrease in *AMI* suggests the stagnation of economic development, as depicted in Figure 3. So even though the Beijing economic system grew exponentially in overall size, it did not achieve higher levels of development from an ENA perspective.

Compared to other types of systems, economic networks exhibit lower *AMI*. For example, *AMI* is 1.38 bits for the water flow network of Sarmato in northern Italy [24] and 1.336 bits for the Cone Spring energy network [34]. By contrast, *AMI* for world commodity trade from 1986 to 2001 fluctuated between 0.56 and 0.74 nats [32]. The *AMI*s calculated here were even lower and varied between 0.45 and 0.58 bits from 1985 to 2010. It should be noted that economic systems are more complicated than other types of systems in that they possess bidirectional flows between many nodes, especially when the number of nodes is fewer than 12. This is not always true for other systems, however. For example, energy flows predominately from lower to higher trophic levels in ecosystems. While energy can flow directly from a rabbit to a tiger, it does not flow in the opposite direction.

### 3.3. Ascendency trend and analysis of growth and development

**Figure 4:**
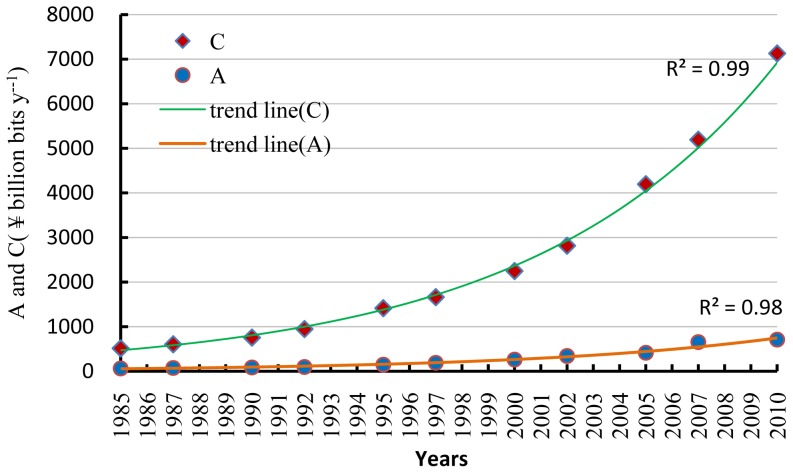
Ascendency (*A*) and development capacity (*C*) for Beijing over the study period.

Ascendency has been identified with an increase in network size and organization; therefore, increases in ascendency can be translated into growth and development. Everything that grows and develops is constrained by temporal, spatial, or material factors. Nothing can grow and develop without bounds. Development capacity represents the upper bound for ascendency. We substituted flow data for each currency network into Eqs. (8) and (9) to track the ascendency and development capacity for each year. (The *TST*s in Eqs. (8) and (9) have also been adjusted by GDP index for inflation.) The results are depicted in Figure 4. During 1985–2010, both ascendency and development capacity increased exponentially. The former increased from ¥63 billion bits y^−1^ to ¥710 billion bits y^−1^, with exponential growth rate 10.20%, whereas the latter increased from ¥514 billion bits y^−1^ to ¥7127 billion bits y^−1^, with exponential growth rate 11.09%. Ascendency accounted for less than 13% of the development capacity in each year of the study period. Similarly, Kharrazi et al. reported that ascendency accounted for less than 20% of development capacity for all six economic resource trade flow networks that they followed [32]. It would appear from these two studies that the room for growth and development in each case remains large.

While we have analyzed the state of growth and development for Beijing, the results do not reflect the proportionate roles of growth and development in the observed change. The separate factors, growth and development possess different physical dimensions. The *TST* factor has units of ¥billion y^−1^, while the *AMI* is measured in bits. We, therefore, invoked the complete decomposition model (described above in the Methods Section) to distinguish the proportional contributions of *TST* and *AMI* to ascendency. During 1985–2010, *TST* increased exponentially, while *AMI* fluctuated marginally. *TST*, therefore, had a continuously positive effect on ascendency during that time, whereas *AMI* did not always stimulate an increase in ascendency. According to the *AMI* fluctuations, the years between 1985 and 2010 can be classified into six stages (Table 1). Within each stage, *AMI* increased or decreased uniformly. We substituted *TST* and *AMI* for each stage into Eqs. (12) and (13) to obtain the contributions of *TST* and *AMI*, respectively, to ascendency. The results in Table 1 show that the contribution of *TST* to increasing ascendency was more than 80% during each stage, except for the fifth. In the second, fourth and sixth stages, the contributions of *AMI* to increasing ascendency were negative. In the remaining stages, the contributions of *AMI* to ascendency were positive. These results confirm that growth is the primary characteristic of the Beijing economy.

**Table 1:** Contribution of TST and AMI to ascendency in Beijing.

Contribution	1985–1987	1987–1992	1992–2002	2002–2005	2005–2007	2007–2010
TST (%)	90.9	179.8	82.1	185.9	56.3	352.3
AMI (%)	9.1	−79.8	17.9	−85.9	43.7	−252.3

### 3.4. Contributions of direct flows to ascendency and suggestions for sustainable development

Growth and development are key features of sustainable development. Analyzing the contribution of each direct flow to ascendency could suggest particular clues as to how a more sustainable system might be achieved. The most recent year (2010) is perhaps the most relevant network with which to analyze the effect of direct flows on ascendency. In Eq. (8), each flow contributes one and only one term to the summation that defines ascendency. We, therefore, calculated the contributions corresponding to each individual flow in Figure 1 and listed them in Table 2. Of the 64 terms, 33 are negative, which means that almost 50% of the direct flows have a negative effect on system growth and development. Such a large proportion of negative contributions possibly owes to the high degree of aggregation necessary to condense 40 or so sectors into only six. (Aggregation of nodes inflates the prevalence of parallel [redundant] flow pathways.) Maximum contribution to ascendency was observed for inflows to industry and minimum contributions were from industry sales to other services. From the supply side, other services contributed 18.6% to total ascendency, inflows 34.6%, and value-added capital 23.8%. Thus, inflows and value-added capital together contributed almost 60%. From the demand side, industry accounted for 26.9% of ascendency, and final demand and outflow contributed 26.1% and 26.0%, respectively. Thus, the total contribution by final demand and outflows amounted to more than 50%. Regardless of whether reckoned from the supply or the demand side, the main contributions to ascendency involve the exchange of a commodity or service with other systems.

**Table 2:** Contribution of each direct flow to ascendency in 2010 (¥ billion bits y^−1^).

	Agriculture	Industry	Construction	TPT	CD	Other sevices	Final demand	outflow	Supply sums	Proportion (%)
Agriculture	2.7	−1.5	−0.2	−0.0	−0.3	−1.2	13.2	−1.3	11.4	1.6
Industry	−1.7	21.5	13.8	−9.4	−14.0	−34.4	−19.1	94.6	51.3	7.2
Construction	−0.0	−1.3	−1.5	−1.3	−2.1	−5.6	75.2	7.8	71.3	10.0
TPT	−0.4	−16.0	−2.5	24.4	−2.1	−2.4	6.9	17.6	25.5	3.6
CD	−0.4	−3.5	1.6	−1.9	−3.1	5.4	−3.4	9.3	4.0	0.6
Other services	−1.2	−30.6	−6.6	−11.3	2.2	10.7	112.4	56.4	131.9	18.6
Value added	0.3	−20.4	0.3	25.9	42.4	120.7	0.0	0.0	169.2	23.8
Inflows	11.0	243.1	7.1	4.5	8.3	−28.4	0.0	0.0	245.5	34.6
Demand sums	10.2	191.2	11.9	30.9	31.4	64.8	185.2	184.3	710.0	
Proportion (%)	1.4	26.9	1.7	4.3	4.4	9.1	26.1	26.0		

TPT represents transportation, post and telecommunication.CD represents commercial and diet.

The contributions of each sector to ascendency are dual. For example, industry contributed only 7.2% to total ascendency from the supply side but 26.9% from the demand side. Such difference exists despite the fact that supply and demand are equal for each sector. This points out that the roles of supply and demand are different for each sector. Overall ascendency, however, remains symmetric with respect to supply and demand. Overall ascendency, therefore, appears to be an unbiased criterion with which to evaluate the growth and development of an economy.

To achieve sustainable development, it is not always advisable to use growth and development as the sole desideratum. Rather, from a holistic viewpoint, sustainability requires a balance between efficiency (ascendency) and resilience [21]. Systems with either vanishingly small ascendency or insignificant resilience inevitably perish. A system lacking ascendency has neither the extent of activity nor the internal organization necessary for survival. Conversely, systems that are tightly constrained and efficiently honed to a particular environment are prone to collapse in the face of even minor novel disturbances. Only when the balance between ascendency and resilience lies in a suitable range can a system persist. Available data indicate that an ecosystem is near its optimal sustainable state when the ratio of ascendency to development capacity is about 0.401 (i.e. ascendency/resilience ratio of 0.67) [41]. The closer the ascendency/resilience ratio is to 0.67, presumably the more sustainable is that system. For a ratio greater than 0.67, pathways contributing greatest to ascendency should be weakened by appropriating fewer resources to them. For a ratio less than 0.67, the same pathways making the greatest contributions should be strengthened to move the system toward greater sustainability.

There is no reason, however, to suppose that economic systems and ecosystems have the same optimal sustainable state. For one, currency networks are more complicated than ecosystem networks, so that the value of 0.67 may not indicate the optimal sustainable economic state. According to Figure 4, the ratio of ascendency to development capacity is less than 0.13 (i.e. the ascendency/resilience ratio is less than 0.15 [and far less than 0.67]). Although we cannot ascertain the optimal sustainable state for economic systems, an extremely low ascendency suggests that its enhancement is probably desirable in order to draw nearer to sustainable development.

To increase ascendency, one can either increase *TST* or *AMI* (or both, if possible). *TST* can be increased by absorbing more resources. If resources are limiting, the best way to augment ascendency is to increase *AMI* by reapportioning flows within the network. This should involve strengthening those pathways with higher positive contributions to ascendency and weakening those having the most negative effects. For example, the economic ascendency of Beijing would be fostered if industry focused more on augmenting its external inflows and less on its demands from other services. Of course, when adjusting flows in the network, factors such as market demand, resource availability, and price must also be considered.

## Summary and Conclusions

I-O tables for Beijing during 1985–2010 were converted into currency networks, the growth and development of the Beijing economy were analyzed using ENA, and suggestions for sustainable development were proposed:

(1) During 1985–2010, the Beijing economic system grew exponentially in overall activity; however, the system did not develop uniformly, but rather fluctuated, mostly due to external factors such as global economic crises. From the perspective provided by ENA, growth is the major characteristic of the Beijing economy and no clear trend in development is evident.

(2) If sustainability in ecosystems is an appropriate analog to that in economics, it would appear necessary to enhance the ascendency of the Beijing economy. This can be achieved in either of two ways. One is to strengthen pathways with positive contributions to ascendency and the other is to weaken pathways having negative effects. Efforts at remediation should begin with those pathways contributing terms with the greatest absolute magnitudes.
